# Isolated Volar Dislocation of the Distal Radioulnar Joint Treated With Successful Closed Reduction

**DOI:** 10.7759/cureus.15656

**Published:** 2021-06-15

**Authors:** Matthew T Glazier, Hayden B Schuette, Benjamin A Schnee, Brian Skura, Craig Goubeaux

**Affiliations:** 1 Orthopedic Surgery, OhioHealth, Columbus, USA; 2 Orthopedic Surgery, Florida Orthopedic Institute, Tampa, USA

**Keywords:** volar druj dislocation, wrist pain, druj injury, isolated druj injury, reduced wrist rotation, succesful closed reduction of druj dislocation, volar druj injury

## Abstract

We report the case of A 34-year-old right-hand-dominant male who presented with an isolated left volar dislocation of the distal radioulnar joint (DRUJ) without any associated fractures. The patient had sustained the injury in an altercation in the evening prior to the presentation and had woken up the next morning with left wrist pain and restricted wrist motion. Closed reduction was successful under conscious sedation and the patient was treated conservatively with splint immobilization without needing operative intervention.

This report highlights a rare injury pattern - an isolated volar DRUJ dislocation - that was successfully closed reduced, despite reports that this injury pattern frequently requires open reduction.

## Introduction

An isolated volar dislocation of the distal radioulnar joint (DRUJ) is a rare injury pattern that was first described by Desault in 1777 [1]. DRUJ dislocations are often dorsal, and instability presents with an associated injury to the triangular fibrocartilage complex (TFCC) or an associated forearm fracture [2]. Following acute volar dislocations of the DRUJ, patients typically present with diminished forearm rotation and a loss of prominence of the ulnar head. Patients can also present with the wrist joint locked in supination [3]. A literature review has revealed several case reports of irreducible acute DRUJ dislocations [4-6]; however, there is limited literature describing reducible acute DRUJ dislocations with no associated fracture. We present a case of a patient with an acute volar DRUJ dislocation and no associated fracture, which was successfully reduced in the emergency department.

Informed consent has been obtained from the patient for the publication of the details of the case.

## Case presentation

A 34-year-old right-hand-dominant male presented to the emergency department with acute left wrist pain and diminished forearm rotation. The patient stated that he had been in an altercation the evening prior and had woken up the next morning without any recollection of the mechanism of injury. He had no relevant previous medical or surgical history. He denied any previous injuries to his left wrist or forearm.

On physical examination, the patient complained of pain over the ulnar aspect of the left wrist. The dorsal prominence of the ulnar head was no longer appreciated and was instead palpable volarly. There were no open skin lacerations or wounds. The patient was neurovascularly intact with the appropriate function of the anterior interosseous, posterior interosseous, and ulnar nerves as evident by intact cardinal hand movements. The wrist was mildly swollen, and passive and active range of motion was limited.

**Figure 1:**
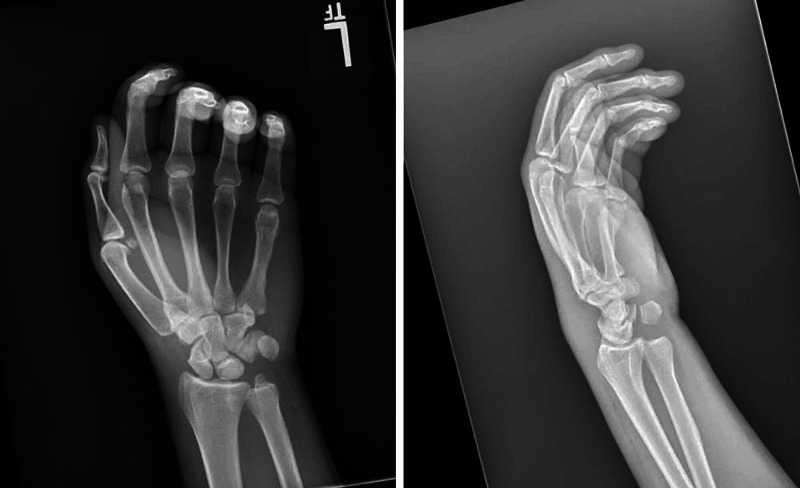
Initial posteroanterior (left) and lateral (right) radiographs of the injury

Initial radiographs were obtained (Figure 1), which demonstrated abnormal overlap of the distal ulna with respect to its normal articulation with the distal radius on posteroanterior view and clear volar dislocation of the ulna on the lateral view. Additionally, left forearm and elbow radiographs demonstrated no fractures or dislocations.

**Figure 2:**
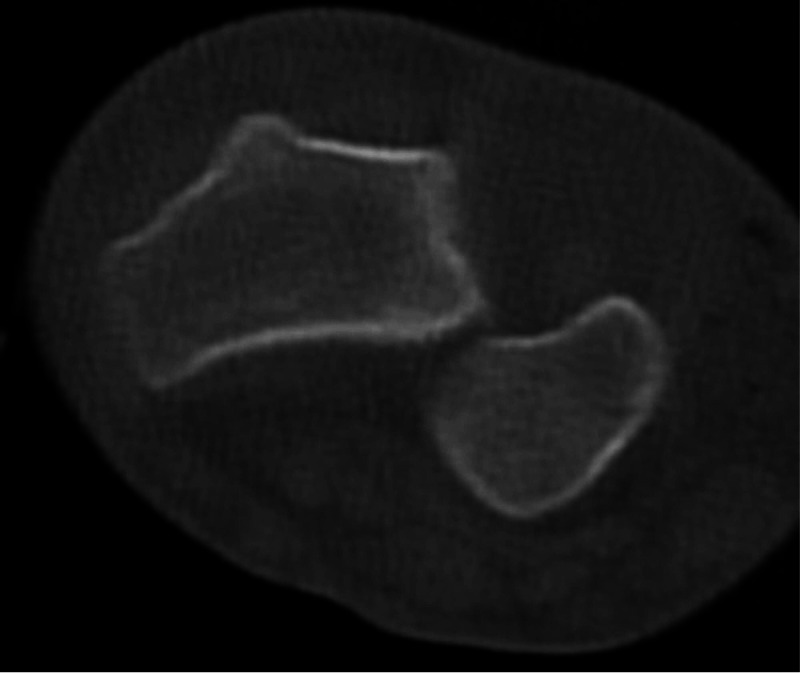
Axial cut CT scan at the level of the distal radioulnar joint CT: computed tomography

**Figure 3:**
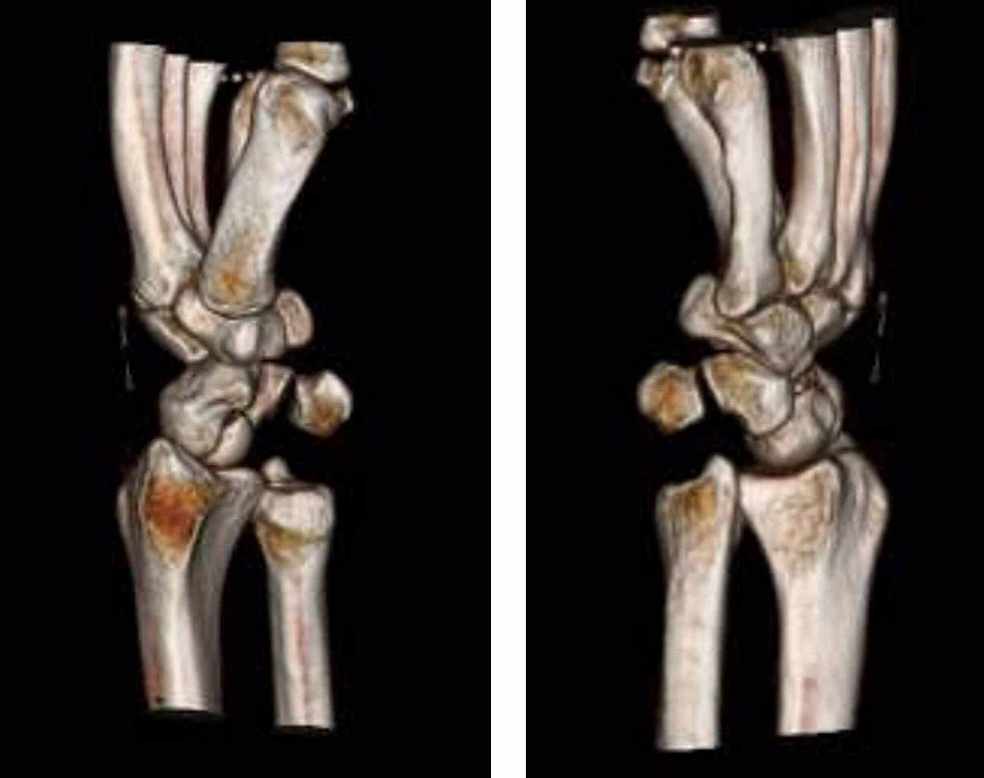
Three-dimensional CT reconstruction of the left wrist CT: computed tomography

A CT scan was also obtained to look for any associated injuries or fractures. CT confirmed the isolated volar dislocation of the ulnar head from the distal radial sigmoid notch. This was best seen on the axial cut at the level of the DRUJ (Figure 2). A 3D reconstruction of the scans was also performed to obtain additional information on the injury and aid with closed reduction and treatment (Figure 3).

**Figure 4:**
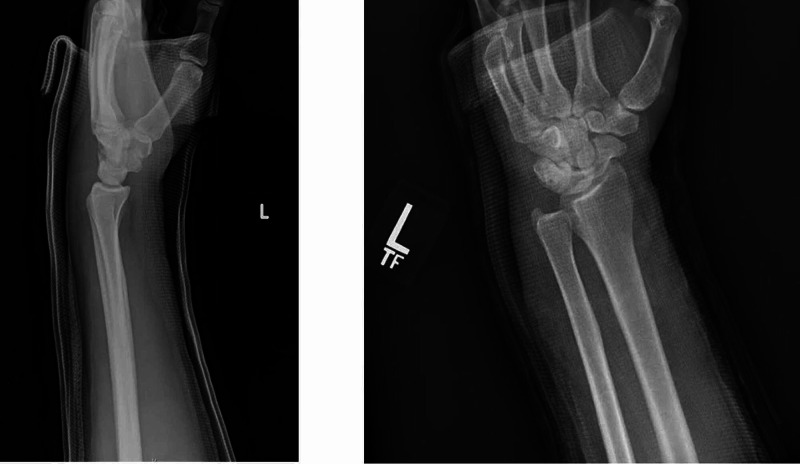
Post-reduction radiographs including lateral (left) and posteroanterior (right) views

After receiving informed consent from the patient, conscious sedation was performed with the assistance of the emergency room provider. Closed reduction of the left volar DRUJ dislocation was successful by applying slight distraction to the radius and ulna and applying a dorsally directed force to the ulnar head. As the wrist was slightly pronated with continuous direct pressure being maintained, an audible click was heard, and reduction was appreciated. Once the DRUJ was reduced, the wrist was passively taken through the full normal range of motion including flexion, extension, and pronosupination. Post-reduction stability was assessed throughout motion and no instability was noted. Range of motion and stability was comparable to the contralateral uninjured wrist after reduction. The patient was immobilized in neutral forearm rotation and 90 degrees of elbow flexion using a well-molded sugar tong fiberglass splint. Post-reduction radiographs were obtained and confirmed the successful reduction of the DRUJ (Figure 4).

**Figure 5:**
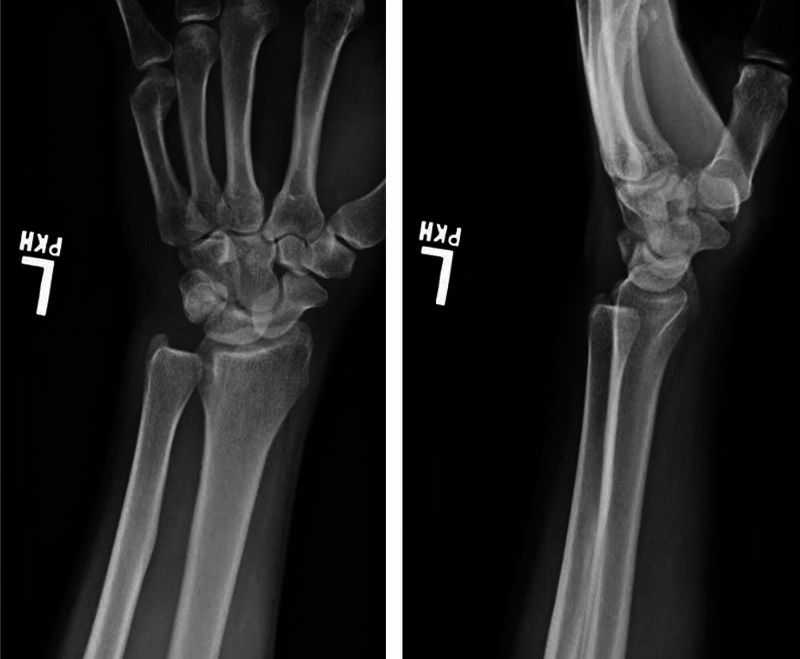
Posteroanterior (left) and lateral (right) radiographs taken at follow-up demonstrating maintenance of reduction without recurrence of volar ulnar dislocation

The patient was seen for a follow-up in the orthopedic clinic the subsequent week, and repeat radiographs of the left wrist demonstrated a maintained reduction of the DRUJ. The patient's pain had improved, and he was instructed to keep the wrist immobilized for another four weeks. Two weeks later, the patient presented once again to the emergency department after his original fiberglass splint had got wet and loosened, requiring a new splint to be applied. Repeat radiographs again showed a concentric reduction. The patient’s splint got wet and loosened once again at six weeks from the date of his original injury, and he was transitioned to a prefabricated removable wrist splint. At that time, his pain had continued to improve and he had full range of motion of the left wrist. The radiographs from the outside emergency department during this last encounter demonstrating maintenance of reduction without recurrence of volar ulnar head dislocation are presented below (Figure 5). Unfortunately, this patient was lost to long-term orthopedic follow-up due to a period of incarceration, but he has had subsequent medical encounters within our health system nearly three years after his original injury without noted residual left wrist pain, diminished range of motion, or any additional wrist complaints, and a satisfactory clinical recovery can be inferred.

## Discussion

This case presented a unique injury pattern - an isolated volar DRUJ dislocation - that was successfully closed reduced in the emergency department. This patient was able to avoid an open reduction and was successfully treated instead with a short duration of immobilization. Usually, DRUJ dislocations require surgical interventions with open reduction as well as internal fixation of associated fractures.

When a DRUJ dislocation is clearly evident, as was the case in our patient, associated injuries should be suspected. DRUJ dislocations are often associated with distal radius fractures with an associated ulnar styloid fracture, especially if the ulnar styloid fragment is displaced by greater than 2 mm, or with Galeazzi type fractures [7,8]. Physicians should also maintain a heightened suspicion for DRUJ dislocations when dealing with fractures causing acute radioulnar length discrepancies such as a distal radius fracture with a significant amount of shortening, both bone forearm fractures, or any fractures with extension into the DRUJ. Another associated injury that may lead to DRUJ instability is a complete tear of the interosseous membrane with an associated radial head fracture, also known as an Essex-Lopresti fracture [5]. Conversely, it is important to have a high suspicion for DRUJ instability when a patient presents with wrist pain and no apparent fractures, as was the case with our patient. This is a commonly missed injury and, in particular, volar DRUJ dislocations may be initially missed out in as many as 50% of patients who present to the emergency department [9]. When missed, this can lead to chronic instability and pain.

The stability of the DRUJ is provided by static bony and ligamentous restraints as well as dynamic muscular restraints [2]. Radioulnar ligaments are the primary ligamentous stabilizers of the DRUJ. Secondary stabilizers of the DRUJ include the pronator quadratus muscle and extensor carpi ulnaris tendon and sheath, interosseous membrane, DRUJ capsule, and articular disk [10]. In volar dislocations, the dorsal radioulnar ligament and volar joint capsule are disrupted. Gross instability of the DRUJ generally requires disruption of multiple structures, and complete dissociation does not occur unless there is tearing of the interosseous membrane [10]. 

During this particular patient encounter and unlike most case reports, closed reduction was successful on the first attempt with slight distraction and a dorsally directed force applied to the ulnar head. More often, a volar DRUJ dislocation is irreducible and requires open treatment and reduction [4-6]. A recent case report described a successful closed reduction maneuver for a volar dislocation with an impaction fracture of the ulnar head on the radius [11]. An assistant applied compressive pressure to the interosseous membrane of the forearm using the palms of both hands while the physician was able to reduce the ulnar head with dorsally directed pressure once the ulnar head was disengaged from the radius. This was not completely new as Cotton and Brickley [12] had described the “Boyer method” in 1912, where fingers were placed directly between the radius and ulna to distract the bones, which aided in reduction. In another case, the closed reduction could be performed only once the patient was under general anesthesia and had received full muscle relaxation [3]. While these similar maneuvers enabled successful closed reduction, those were not necessary in our case, as there was no impaction of the ulnar head on the radius or block to initial reduction.

Closed reduction of these injuries can be complicated by some well-documented blocks to reduction. These include impaction of the ulnar head on the rim of the sigmoid notch of the radius [5], spasm of the pronator quadratus [3,13], displacement of the ulnar styloid that leads to extensor carpi ulnaris dislocation, entrapment of extensor digitorum communis tendon to the ring and little finger, extensor digiti minimi tendon, flexor pollicis longus, fragments of the TFCC, extensor retinaculum, and even the median nerve [2,3].

When the DRUJ is stable after successful closed reduction of an isolated dislocation without associated fractures, treatment in a well-molded above-elbow cast or splint for three to four weeks followed by a transition to a short arm cast or splint for an additional two to three weeks is appropriate [10]. Interval radiographs are recommended to ensure successful maintenance of the reduction. Joint reduction following volar dislocations of the DRUJ is traditionally more stable with the wrist in pronation, whereas dorsal dislocations have increased stability in supination [10]. Similar to our case, Larrivée et al. have reported successful closed reduction and splinting of a volar DRUJ dislocation; however, their patient had an associated ulnar impaction fracture [11].

## Conclusions

Isolated volar dislocations of the DRUJ are rare injuries that physicians need to be aware of when patients present with wrist pain and diminished forearm rotation. Closed reduction is feasible under conscious sedation with slight distraction and constant pressure to reduce the ulnar head, although physicians should be aware that open reduction is frequently required. Satisfactory clinical results can be seen with a short duration of immobilization in a cast or splint if the DRUJ remains stable after closed reduction.
